# Laboratory and Field Testing of an Automated Atmospheric Particle-Bound Reactive Oxygen Species Sampling-Analysis System

**DOI:** 10.1155/2011/419476

**Published:** 2011-03-24

**Authors:** Yungang Wang, Philip K. Hopke, Liping Sun, David C. Chalupa, Mark J. Utell

**Affiliations:** ^1^Center for Air Resource Engineering and Science, Clarkson University, Potsdam, NY 13699-5708, USA; ^2^Department of Environmental Medicine, University of Rochester Medical Center, Rochester, NY 14627, USA

## Abstract

In this study, various laboratory and field tests were performed to develop an effective automated particle-bound ROS sampling-analysis system. The system uses 2′ 7′-dichlorofluorescin (DCFH) fluorescence method as a nonspecific, general indicator of the particle-bound ROS. A sharp-cut cyclone and a particle-into-liquid sampler (PILS) were used to collect PM_2.5_ atmospheric particles into slurry produced by a DCFH-HRP solution. The laboratory results show that the DCFH and H_2_O_2_ standard solutions could be kept at room temperature for at least three and eight days, respectively. The field test in Rochester, NY, shows that the average ROS concentration was 8.3 ± 2.2 nmol of equivalent H_2_O_2_ m^−3^ of air. The ROS concentrations were observed to be greater after foggy conditions. This study demonstrates the first practical automated sampling-analysis system to measure this ambient particle component.

## 1. Introduction

Substantial efforts are being made to elucidate the mechanisms of adverse human health effects by airborne particulate matter (PM). Fine particles (PM_2.5_) have been found to be correlated with cardiopulmonary morbidity and mortality [1]. Ultrafine particles (UFPs, *D*_*p*_ < 100 nm) have been associated with effects in animals [2, 3] and humans [4, 5]. However, the chemical components of the particles that drive the mechanisms resulting in health effects are not yet well understood. Since oxidative stress is thought to be a critical factor in driving health effects [1], it is essential to identify and link specific oxidative particulate components, such as reactive oxygen species (ROS).

ROS include oxygen-containing compounds with strong oxidative capacity. Molecules like H_2_O_2_, organic peroxides, and nitrite peroxides, ions like hypochlorite ion (OCl^−^) peroxide anion (O_2_^−^), and radicals like hydroxyl (^∙^OH) and superoxide radicals (^∙^O_2_^−^), and organic peroxyl (ROO^∙^) are all grouped as “reactive oxygen species”. ROS can be generated endogenously during the cell metabolism through reaction of the inhaled PM components such as metals (Fe, Cu, and Zn) and polycyclic aromatic hydrocarbon (PAH) [6, 7]. The excess oxidative stress from the ROS leads to lipid peroxidation, DNA damage, and protein oxidation, and has been implicated in the increased incidence of cardiopulmonary disease, asthma, and chronic obstructive pulmonary disease [8–11]. Recently, ROS was found to be present in PM, especially in the UFPs component [12, 13]. These particle-bound ROS are believed to induce effects on human health analogous to that of endogenous ROS. 

The major sources of particle-bound ROS in the atmosphere are reaction between volatile organic compounds (VOC) and oxidants such as ozone (O_3_) or hydroxyl radicals (OH). For example, the oxidation products of biogenic VOC and O_3_ have low vapor pressure and can easily condense on the surface of existing PM or nucleate to form secondary organic aerosols (SOA). These components also include peroxides and radical species that constitute some of the particle-bound ROS [14, 15]. In principle, photochemical reactions generate the majority of free radical species in the atmosphere during the daytime. Without sunlight, the particle-bound ROS formation mechanism is largely influenced by the NO_3_ radical [16] and the OH radical, the latter of which was formed from the ozone and alkene reactions [17]. The specific route through which atmospheric particle-bound ROS are formed remains unclear.

Efforts have been made to characterize the ambient particle-bound ROS. The photochemical intensity was a major factor affecting ROS concentrations in smaller particles, especially in UFPs [18]. The concentration of tropospheric hydroxyl radicals can be described by a linear dependence on solar ultraviolet radiation [19]. Hydroperoxides were simultaneously measured in both gas and aerosol phases, and about 40% of particle-bound H_2_O_2_ were associated with PM_2.5_ [20]. Concentration data on atmospheric ROS in the particle phase are limited and reported in the unit of nmol of equivalent H_2_O_2_ m^−3^ of air [12, 13, 18, 21, 22].

In prior studies, filters were commonly used to manually collect particle-bound ROS. ROS was then extracted from the filters and analyzed using the 2′  y7′-dichlorofluorescin (DCFH) fluorescence technique in the laboratory. This method might underestimate ROS concentrations because the short lived species may be more chemically active than the components measured days or weeks later. The method is quite labor intensive [23]. The lack of suitable methods to routinely sample and immediately analyze ROS in the field has restricted the evaluation of the health effects of particle-bound ROS. 

A continuous, automated particle-bound ROS system was previously developed [23]. DCFH was employed as a general, nonspecific indicator of particle-bound ROS concentration. A sharp cut cyclone and a particle-into-liquid-sampler (PILS) were used to collect PM_2.5_ into aqueous slurry that contained the DCFH solution. The fluorescent intensity (FI) was then measured with a flow-through fluorescence detector. Quantification was obtained by relating the sample's FI to that of an equivalent concentration of H_2_O_2_. This initial laboratory system was not deployed because of uncertainties in its operation in the field. Issues of concern included the stability of the reagent solutions under field conditions and the complexity of the design. The current study presents the results from the laboratory testing of a modified system and measurement of the solution stabilities leading to field measurements of atmospheric particle-bound ROS concentrations in Rochester, NY.

## 2. Experimental

### 2.1. Instruments

**Figure 1:**
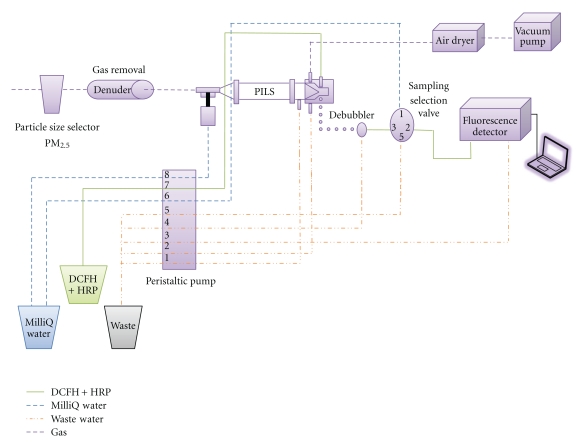
Schematic diagram of the particle-bound ROS automated system.

A schematic diagram of the automated sampling-analysis system is shown in Figure 1. The detailed design and construction of the system were introduced in the previous study [23]. During the optimization and laboratory testing of the system, the membrane reactor and superserpentine reactor were found not to significantly improve the reaction among the DCFH, horseradish peroxidase (HRP) and ROS. Therefore, they were removed from the system and the HRP was directly dissolved into the DCFH solution. 

The current system included a PM_2.5_ sharp-cut cyclone, a manganese dioxide (MnO_2_) denuder to remove gas phase oxidants, and a particle-into-liquid-sampler (PILS, Metrohm Inc.) as the inlet system. The solutions are circulated using an 8-channel peristaltic pump through a selection valve, and a fluorescence detector (FP2020, Jasco Inc.). The sample and blank cycles were run for 3 minutes and 7 minutes, respectively, via the selection valve to eliminate effects of one cycle on the next. To minimize variability arising by visible and long-wavelength UV radiation, as well as to prevent photo-oxidation of the DCFH, the flow lines were covered with aluminum foil. The sampling flow rate was 16.7 L/min.

### 2.2. Reagents

Two solutions, DCFH with HRP and H_2_O_2_ standards, were prepared in a dark environment before the measurements. DCFH is a nonfluorescent reagent that becomes fluorescent upon reaction with ROS. Glass containers were wrapped with aluminum foil to prevent exposure to light. All solutions were prepared with high purity water (resistivity: 18.2 MΩ·cm at 25°C, Millipore Corp.). 

**Figure 2:**
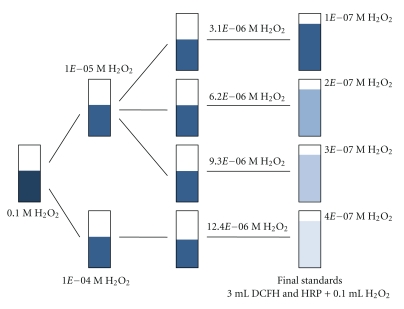
Standard H_2_O_2_ preparation process.

The DCFH and HRP solutions were prepared at 5 *μ*M and 0.5 units/mL, respectively, as described in Appendix A. An standard H_2_O_2_ solution was used to develop the calibration curve. The specific preparation process of H_2_O_2_ standards through a series of dilutions of 30% H_2_O_2_ is shown in Figure 2. Final H_2_O_2_ concentrations of 1 × 10^−7^, 2 × 10^−7^, 3 × 10^−7^, 4 × 10^−7^ M were made by mixing 0.1 mL of intermediate H_2_O_2_ solutions of 3.1 × 10^−6^ M, 6.2 × 10^−6^ M, 9.3 × 10^−6^ M, and 12.4 × 10^−6^ M with 3 mL DCFH solution prepared with HRP. Standard curves were developed from measuring the FI of these final four concentrations of H_2_O_2_.

### 2.3. Procedure

**Figure 3:**
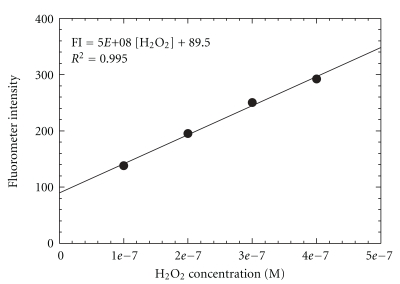
Calibration plot of the system with standard H_2_O_2_ solutions in the field.

The standard operation procedure for running the automated ROS system is given in Appendix B. Calibration of the system was performed with standard H_2_O_2_ solutions of concentrations ranging from 100 to 400 nM, prepared by serial dilutions of a 30% stock solution of H_2_O_2_, with MilliQ water serving as a blank. A HEPA filter was placed in front of the system during calibration running. Figure 3 shows the blank-subtracted linear calibration curve obtained in the field. The system was linear (*R* ^2^ = 0.995) over the range of H_2_O_2_ concentrations by least-squares analysis. The relationship between H_2_O_2_ concentration and FI is expressed as the equation in the figure.

### 2.4. Sampling Location

**Figure 4:**
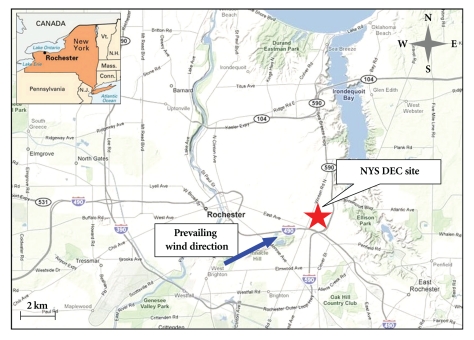
Location of the sampling site in Rochester, NY.

The particle-bound ROS concentrations, O_3_ concentrations and meteorological parameters (ambient temperature, relative humidity, wind direction and speed) were continuously measured during the period of August 12 to 18, 2009 at the New York State Department of Environmental Conservation (NYSDEC) site in Rochester, NY. The site is located at 43°08′46′′ N, 77°32′53′′ W, adjacent to Interstate Highway I-490 and I-590, as well as NY Route 96, a major route carrying traffic traveling to and from downtown Rochester (see Figure 4). 

## 3. Results and Discussion

### 3.1. Stabilities of the DCFH and H_2_O_2_ Solutions

**Figure 5:**
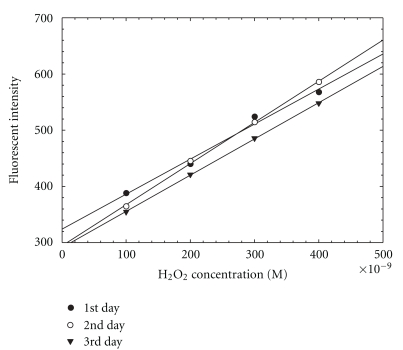
Stability of 5 *μ*M DCFH at room temperature.

**Figure 6:**
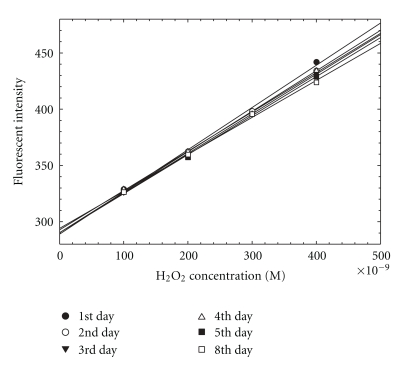
Stability of H_2_O_2_ standard solutions at room temperature.

The stability of the chemical reagents is important for a practical system that can be maintained in the field with a reasonable level of effort. Therefore, the stabilities of DCFH and H_2_O_2_ standards were examined. The experimental stability results for 5 *μ*M DCFH stored at room temperature are presented in Figure 5 and Table 1. It can be seen that 5 *μ*M DCFH was stable for three days at room temperature. The stability of the H_2_O_2_ standards is shown in Figure 6 and Table 2. The solutions can be kept at room temperature for up to eight days. These results provide the feasibility in the field deployment of the automated sampling-analysis system since the unit does not require daily solution preparation.

### 3.2. Laboratory Testing of the System

**Figure 7:**
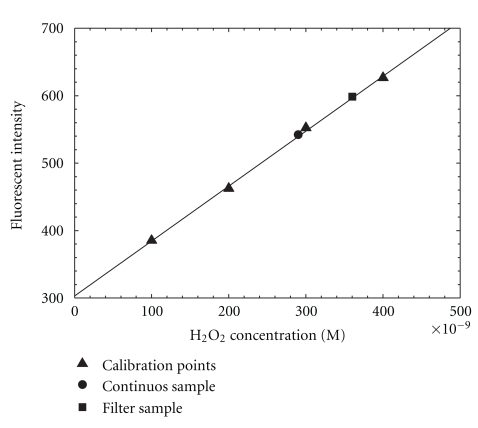
Laboratory test of the automated ROS sampling-analysis system.

Laboratory tests were performed by sampling particle-bound ROS from an *α*-pinene-ozone generator [24] for 30 minutes at a flow rate of 16.7 L min^−1^. The continuous sample and filter sample were compared with H_2_O_2_ standard solutions (see Figure 7). During a 30-minute sampling period, the FI was constant. The filter point represents sample taken on a baked quartz filter for 15 minute intervals. This sample duration limits the loss of short lifetime ROS. 50 mL of 5 *μ*M DCFH was added to the filter sample and the filter was then sonicated for another 15 minutes. The FI of the filter particle-bound ROS was comparable to that measured with the continuous system. The FI results of filter and continuous samples were plotted in the standard calibration curve shown in Figure 7.

A somewhat higher FI was obtained from the filter sample, which contradicts the assumption that the filter sampling method may result in the loss of short lifetime ROS, leading to lower FI in filter sample than from continuous system sample [23]. The 15-minute extraction of the filter sample probably increased the extent of DCFH oxidization rather than decreased short lifetime ROS. Another possible reason was that the extraction volume of DCFH solution was 50 mL, which was larger than the volume used for the continuous system sample (10 mL). Therefore, higher FI for filter particle-bound ROS was produced. After the chemical reagents stability check and laboratory performance testing, the automated particle-bound ROS sampling-analysis system was ready for field testing.

### 3.3. Field Testing of the System

Table 3 summarizes statistics of meteorological parameters. Persistently sunny and humid weather (average ambient temperature: 25.75°C, average relative humidity: 66.17%) was given by Ontario Lake seated to the north. The prevailing winds during this period were mainly from the southwest with an average wind speed of 1.44 m/s. During the seven days of study, there was one foggy day (6:00–9:00 AM on August 13) and two rainy days (precipitation less than 0.4 cm and lasted for only five minutes).

**Figure 8:**
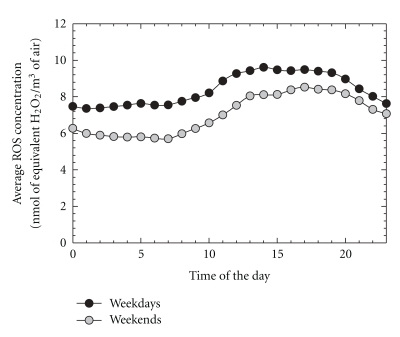
Diurnal variations of mean particle-bound ROS concentrations measured on weekdays and weekends, respectively.

Figure 8 shows the diurnal variations of hourly average particle-bound ROS concentrations measured on both weekdays and weekend days. The weekday concentrations were generally higher than those measured on weekends. The greatest difference was observed during early morning when primary emissions from motor vehicles operating on nearby highways (I-490 and I-590). There were significant differences in traffic volumes between weekdays and weekend days. The highest average ROS concentrations occurred during the afternoon. The daytime ROS concentrations were slightly greater than the nighttime levels on both weekdays and weekends. 

Similar results have been found in Rubidoux, CA, and New York City, where the particle-bound ROS did not drop as much as the O_3_ concentrations during nighttime [12, 13]. New formation through the NO_3_ pathway and the transported longer-lived ROS play important roles in the elevated nighttime ROS concentrations. The nitrate radical reactions along with the oxidation of alkenes by the residual ozone led to ROS concentrations that were only slightly lower than the daytime concentrations [22]. These diurnal patterns suggest that photochemical reactions and vehicular emissions are the main sources of the atmospheric particle-bound ROS in urban areas. 

Table 4 compares the particle-bound ROS concentrations measured in different urban locations with filter collection and extraction methods. Except for the flushing, NY study, all of the studies were conducted during the summer. The overall average ROS concentration from all the studies was 6.1 nmol m^−3^. The lowest ROS concentration (0.54 nmol m^−3^) was measured in Taipei, Taiwan, which was an order of magnitude lower than the ROS concentrations in the other studies. Short-lived ROS with lifetime less than 3-hr cannot be estimated, since 3-hr samples were collected in that study [22]. Thus, concentrations of ROS with lifetime less than 3-hr might be greatly underestimated. The average particle-bound ROS concentration of 8.3 ± 2.2 nmol m^−3^ measured in this study is among the typical values reported for the urban sites of U.S. and Asia.

**Figure 9:**
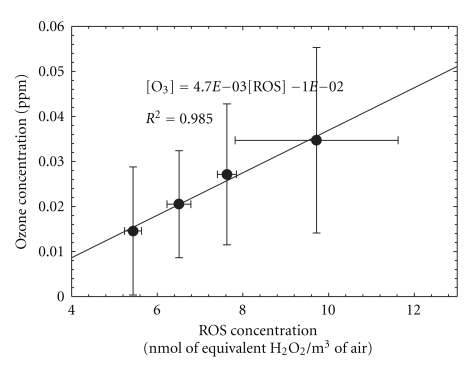
Correlation between mean ozone concentrations and mean particle-bound ROS concentrations (error bars represent standard deviations).

Table 5 summarizes the Pearson correlation coefficients between the hourly averaged ROS values, the other measured pollutants, and the meteorological variables. All the variables were measured at the same site and were averaged to hourly values. Details of the measurements are described elsewhere [25–27]. The scatter plot of the average ozone concentrations and the corresponding ROS concentrations for the entire sampling period is shown in Figure 9. The ozone concentrations, measured as a potential indicator of the intensity of photochemical reactions [18], were obtained from standard photometric ozone monitors maintained by the NYSDEC at this location. The statistically high correlation (*r* ^2^ = 0.985) between ozone and ROS concentrations indicates that the formation of ROS is strongly influenced by photochemical activity, consistent with the previous studies [13, 18, 22]. 

The largest standard deviation of ROS concentration was found for the highest level shown as the top point. It was due to the higher ROS concentrations measured on the August 13, a foggy morning, with an average ROS concentration of 12.31 nmol m^−3^. This event may have resulted from rapid uptake of water-soluble oxidants into the aqueous phase leading to high residual ROS concentration. In addition, the yields of H_2_O_2_ and other complex peroxides were observed to increase substantially in the presence of water vapor in the air from another recent study [28].

## 4. Conclusions

Chemical reagent stability and laboratory performance testing suggested the feasibility of field application of an automated atmospheric particle-bound ROS sampling-analysis system. Sampling of summertime ambient ROS was successfully performed for seven days in Rochester, NY. The average ROS concentration of 8.3 ± 2.2 nmol m^−3^ is among the typical values reported for the urban sites in the U.S. and Asia. It was also found that photochemical reactions and vehicular emissions were two major factors affecting the particle-bound ROS concentrations in urban atmosphere. Nighttime ROS concentrations were only slightly lower than daytime levels. The ROS concentrations were observed to be greater in and after foggy weather conditions than clear days. It is probably because there was uptake or production of oxidants in the aqueous phase and when the water evaporated, it left significant amounts of residual ROS in the atmosphere.

 This study has produced the first practical system to measure this particle component. Uncertainties including the PILS particle capture efficiency, the denuder gas-phase ROS removal efficiency, and the denuder replacement frequency need to be quantified in future experiments. The automated particle-bound ROS sampling-analysis system could conceivably be useful for regulatory communities to control ROS pollution. Further studies are required to use ROS concentrations measured at different locations in different seasons and relate them to human cardiopulmonary diseases. 

## Figures and Tables

**Table 1:** Linear regression results for stability of 5 *μ*M DCFH at room temperature.

Day	Linear regression equation
1	*Y* = 10^7^(0.243 ± 0.021)*X* + (0.131 ± 0.057) *R* ^2^ = 0.986
2	*Y* = 10^7^(0.285 ± 0.007)*X* + (0.016 ± 0.018) *R* ^2^ = 0.999
3	*Y* = 10^7^(0.251 ± 0.003)*X* + (0.003 ± 0.007) *R* ^2^ = 1

**Table 2:** Linear regression results for stability of H_2_O_2_ standard solutions at room temperature.

Day	Linear regression equations
1	*Y* = (0.146 ± 0.007)*X* + (−0.005 ± 0.018) *R* ^2^ = 0.996
2	*Y* = 0.14*XR* ^2^ = 1
3	*Y* = (0.137 ± 0.002)*XR* ^2^ = 1
4	*Y* = (0.136 ± 0.004)*X* + (0.01 ± 0.01) *R* ^2^ = 0.999
5	*Y* = (0.135 ± 0.004)*XR* ^2^ = 0.998
8	*Y* = (0.128 ± 0.004)*X* + (0.015 ± 0.012) *R* ^2^ = 0.998

**Table 3:** Statistical characteristics of hourly averaged meteorological parameters.

	Temp (°C)	RH (%)	WS (m/s)	Precipitation (cm)
Mean	25.75	66.17	1.44	0.00
SD	5.02	17.70	0.69	0.04
Min	17.58	33.64	0.24	0.00
Max	35.98	94.22	3.06	0.38

Temp: ambient temperature; RH: relative humidity; WS: wind speed.

**Table 4:** Summary of previous particle-bound ROS studies.

Location	Concentration (nmol H_2_O_2_/m^3^-air)	Period	Reference
Flushing, NY, USA	0.87 ± 0.18	Jan-Feb 2004	[13]
Singapore traffic	15.10 ± 0.10	Dec 2005	[21]
Singapore ambient	5.71 ± 2.30	Dec 2005 (10 am–1 pm)	[21]
Taipei, Taiwan	0.54 ± 0.40	Jul–Dec 2000	[18]
Rubidoux, CA, USA	5.90 ± 1.70	July 2003	[12, 22]
Rochester, NY, USA	8.30 ± 2.19	Aug 2009	This study

**Table 5:** Summary of the Pearson correlation coefficients.

	*D*_10–50_	*D*_50–100_	*D*_100–500_	BC	Delta-C	O_3_	SO_2_	CO	PM_2.5_	Temp	RH
ROS	−0.15	−0.24	−0.33	−0.30	−0.18	0.21	−0.09	−0.29	−0.28	0.28	−0.31
*D*_10–50_	—	0.46	0.26	0.20	0.27	−0.19	0.41	0.33	0.66	−0.05	0.07
*D*_50–100_	—	—	0.59	0.49	0.50	−0.28	0.35	0.52	0.72	−0.20	0.23
*D*_100–500_	—	—	—	0.53	0.52	0.03	0.22	0.65	0.85	0.01	0.04
BC	—	—	—	—	0.36	−0.72	0.20	0.61	0.32	−0.75	0.74
Delta-C	—	—	—	—	—	−0.53	−0.04	0.70	0.16	−0.39	0.38
O_3_	—	—	—	—	—	—	−0.09	−0.28	−0.21	−0.89	−0.88
SO_2_	—	—	—	—	—	—	—	0.03	0.74	0.03	−0.03
CO	—	—	—	—	—	—	—	—	0.40	−0.22	0.28
PM_2.5_	—	—	—	—	—	—	—	—	—	0.06	−0.05
Temp	—	—	—	—	—	—	—	—	—	—	−0.98

(i) *D*_10–50_, *D*_50–100_, and *D*_100–500_ indicate number concentrations of particles in the size range of 10–50 nm, 50–100 nm and 100–500 nm [25], respectively.

(ii) BC and Delta-C indicate the Aethelometer measurement of particles in the 880 nm wavelength and the difference between 370 nm and 880 nm [26], respectively.

(iii) Temp and RH indicate ambient temperature and relative humidity, respectively.

## Appendices

### A. Preparation of the Reagents

The preparation process of 6 L of final solution is describes below.

- A 1 mM 2′  7′-Dichlorofluorescin diacetate (DCFH-DA) solution was prepared by dissolving 14.619 mg DCFH-DA (Sigma-Aldrich Inc) in 30 mL ethanol and stored without light.
- 120 mL of 0.01M NaOH solution was added to 30 mL of 1 mM DCFH-DA solution to deacetylate DCFH-DA to unstable DCFH. The mixture stayed at room temperature for 30 min to complete the deacetalytion.
- A 25 mM phosphate buffer was prepared by dissolving 4.9762 g disodium hydrophosphate (≥99.0%, Sigma-Aldrich Inc) and 15.1400 g sodium hydrophosphate (≥99.0%, Sigma-Aldrich Inc) in 5.85 L MilliQ water.
- The 150 mL hydrolyzed DCFH solution was neutralized with 5.85 L of 25 mM phosphate buffer of pH = 7.2 that contained 26.4 mg enzyme Horseradish peroxidase (HRP, Type I, 113 unit/mg, Sigma-Aldrich Inc). The 6 L solution contained 5 *μ*M DCFH and 0.5 units/mL HRP.

### B. Standard Operating Procedure for the ROS Monitor

The standard operation procedure for running the automated ROS system is the following.

- Check connections between each unit and all tubing to ensure no leakage.
- Turn on the sampling pump and the air dryer and make sure the sampling flow rate at 16.7 L/min.
- Turn on the peristaltic pump, and set the rotation rate at 35 rotations per minute.
- Turn on the PILS and set the tip temperature at 100°C.
- The PILS steam generator temperature will reach 150°C with the setting temperature at 100°C. Subsequently, turn on the fluorescence detector, and set the gain at 1000, attenuation at 1, response speed at standard, excitation and emission wavelength at 485 and 530 nm, respectively.
- Start the computer, set the sampling period of 3 minutes and rinsing period of 7 minutes.
- Start the “Logger Lite” software (version 1.3.2, Vernier Software & Technology) and build a file to save the data.

## References

[1] Pope CA, Dockery DW (2006). Health effects of fine particulate air pollution: lines that connect. *Journal of the Air and Waste Management Association*.

[2] Kreyling WG, Semmler-Behnke M, Möller W (2006). Health implications of nanoparticles. *Journal of Nanoparticle Research*.

[3] Oberdörster G, Utell MJ (2002). Ultrafine particles in the urban air: to the respiratory tract—and beyond?. *Environmental Health Perspectives*.

[4] Peters A, von Klot S, Heier M (2004). Exposure to traffic and the onset of myocardial infarction. *The New England Journal of Medicine*.

[5] Sioutas C, Delfino RJ, Singh M (2005). Exposure assessment for atmospheric ultrafine particles (UFPs) and implications in epidemiologic research. *Environmental Health Perspectives*.

[6] Squadrito GL, Cueto R, Dellinger B, Pryor WA (2001). Quinoid redox cycling as a mechanism for sustained free radical generation by inhaled airborne particulate matter. *Free Radical Biology and Medicine*.

[7] Stohs SJ, Bagchi D, Bagchi M (1997). Toxicity of trace elements in tobacco smoke. *Inhalation Toxicology*.

[8] Ciencewicki J, Trivedi S, Kleeberger SR (2008). Oxidants and the pathogenesis of lung diseases. *Journal of Allergy and Clinical Immunology*.

[9] Kelishadi R, Hashemi M, Mohammadifard N, Asgary S, Khavarian N (2008). Association of changes in oxidative and proinflammatory states with changes in vascular function after a lifestyle modification trial among obese children. *Clinical Chemistry*.

[10] Kirkham P, Rahman I (2006). Oxidative stress in asthma and COPD: antioxidants as a therapeutic strategy. *Pharmacology and Therapeutics*.

[11] Yang W, Omaye ST (2009). Air pollutants, oxidative stress and human health. *Mutation Research/Genetic Toxicology and Environmental Mutagenesis*.

[12] Venkatachari P, Hopke PK, Grover BD, Eatough DJ (2005). Erratum: "Measurement of particle-bound reactive oxygen species in Rubidoux Aerosols". *Journal of Atmospheric Chemistry*.

[13] Venkatachari P, Hopke PK, Brune WH (2007). Characterization of wintertime reactive oxygen species concentrations in Flushing, New York. *Aerosol Science and Technology*.

[14] Mauderly JL, Chow JC (2008). Health effects of organic aerosols. *Inhalation Toxicology*.

[15] Sakulyanontvittaya T, Duhl T, Wiedinmyer C (2008). Monoterpene and sesquiterpene emission estimates for the United States. *Environmental Science and Technology*.

[16] Wayne RP, Barnes I, Biggs P (1991). The nitrate radical: physics, chemistry, and the atmosphere. *Atmospheric Environment Part A*.

[17] Paulson SE, Orlando JJ (1996). The reactions of ozone with alkenes: an important source of HO_x_ in the boundary layer. *Geophysical Research Letters*.

[18] Hung HF, Wang CS (2001). Experimental determination of reactive oxygen species in Taipei aerosols. *Journal of Aerosol Science*.

[19] Rohrer F, Berresheim H (2006). Strong correlation between levels of tropospheric hydroxyl radicals and solar ultraviolet radiation. *Nature*.

[20] Hasson AS, Paulson SE (2003). An investigation of the relationship between gas-phase and aerosol-borne hydroperoxides in urban air. *Journal of Aerosol Science*.

[21] See SW, Wang YH, Balasubramanian R (2007). Contrasting reactive oxygen species and transition metal concentrations in combustion aerosols. *Environmental Research*.

[22] Venkatachari P, Hopke PK, Grover BD, Eatough DJ (2005). Measurement of particle-bound reactive oxygen species in rubidoux aerosols. *Journal of Atmospheric Chemistry*.

[23] Venkatachari P, Hopke PK (2008). Development and laboratory testing of an automated monitor for the measurement of atmospheric particle-bound reactive oxygen species (ROS). *Aerosol Science and Technology*.

[24] Venkatachari P, Hopke PK (2008). Development and evaluation of a particle-bound reactive oxygen species generator. *Journal of Aerosol Science*.

[25] Wang Y, Hopke PK, Chalupa DC, Utell MJ Long-term study of urban ultrafine particles and other pollutants.

[26] Wang Y, Hopke PK, Rattigan OV, Zhu Y Characterization of ambient black carbon and wood burning particles in urban areas.

[27] Wang Y, Huang J, Zananski TJ, Hopke PK, Holsen TM (2010). Impacts of the Canadian forest fires on atmospheric mercury and carbonaceous particles in northern New York. *Environmental Science and Technology*.

[28] Reeves CE, Penkett SA (2003). Measurements of peroxides and what they tell us. *Chemical Reviews*.

